# 

**Published:** 1886-08

**Authors:** 


### HALL'S JOURNAL OF HEALTH

ESTABLISHED 1854

CONTENTS.

Vol. 33 No. 8

| | PAGE. |
|---|---|
| Special Notice | 225 |
| Presentiments | 225 |
| A Regime of Health | 228 |
| Outing | 226 |
| Faith Cure | 230 |
| Female Disorders | 232 |
| Boracic Acid for Diabetes | 232 |
| Hall's Journal of Health | 233 |
| Anæsthetic Revelations | 233 |
| A Timely Fable | 235 |
| The Sun Healing Movement | 237 |
| Death Attributed to Voudoo Incantations | 241 |
| A Juvenile Story | 241 |
| Stimulating Old Fruit Trees | 242 |
| Prohibited Bliss | 243 |
| Home Department | 246 |
| Miscellaneous Items | 246 |

INDEX TO ADVERTISEMENTS OF HALF A PAGE AND OVER.

| | PAGE. |
|---|---|
| Orange Blossom | 248 |
| Vaginal Injecting and Suction Syringe | 248 |
| Fellows' Hypo-Phosphites | I |
| Lactated Food | II |
| Celerina | III |
| Lactopeptine | IV |
| Castoria | IV |
| Horsford's Acid Phosphate | V |
| Caulocorea | V |
| Medicated Suppositories | VIII |
| Bovinine | 2d page cover |
| Perforated Paper | 3d " " |
| How to Recuperate | 4th " " |

